# New Diphenol and Isocoumarins from the Aerial Part of *Lawsonia inermis* and Their Inhibitory Activities against NO Production

**DOI:** 10.3390/molecules21101299

**Published:** 2016-09-28

**Authors:** Chang-Syun Yang, Hui-Chi Huang, Sheng-Yang Wang, Ping-Jyun Sung, Guan-Jhong Huang, Jih-Jung Chen, Yueh-Hsiung Kuo

**Affiliations:** 1 Department of Chinese Pharmaceutical Sciences and Chinese Medicine Resources, China Medical University, Taichung 404, Taiwan; tim.tim0619@msa.hinet.net (C.-S.Y.); hchuang@mail.cmu.edu.tw (H.-C.H.); gjhuang@mail.cmu.edu.tw (G.-J.H.); 2 Department of Forestry, National Chung-Hsing University, Taichung 402, Taiwan; taiwanfir@dragon.nchu.edu.tw; 3 Agricultural Biotechnology Research Center, Academia Sinica, Taipei 115, Taiwan; 4 Agricultural Biotechnology Center, National Chung-Hsing University, Taichung 402, Taiwan; 5 Graduate Institute of Marine Biotechnology and Department of Life Science and Institute of Biotechnology, National Dong Hwa University, Pingtung 944, Taiwan; pjsung@nmmba.gov.tw; 6 National Museum of Marine Biology and Aquarium, Pingtung 944, Taiwan; 7 Department of Pharmacy, Tajen University, Pingtung 907, Taiwan; jjchen@tajen.edu.tw; 8 Department of Medical Research, China Medical University Hospital, China Medical University, Taichung 404, Taiwan; 9 Department of Biotechnology, Asia University, Taichung 413, Taiwan

**Keywords:** *Lawsonia inermis*, henna, diphenol, isocoumarin, inhibitory activities against NO production

## Abstract

*Lawsonia inermis* Linn (Lythraceae), also known as henna, is a small shrub or tree distributed throughout Taiwan’s Lanyu Island, in North Africa, and in Australia. Its leaves are used as a folk medicine for the treatment of external hemorrhage and fingernail abscesses. Investigation of the ethyl acetate (EtOAc)-soluble fractions from methanol extract of the aerial part of *Lawsonia inermis* has led to the isolation of a new diphenol, (*Z*)-4,4′-(prop-1-ene-1,3-diyl)diphenol (**1**), two new isocoumarin carbonates, inermiscarbonates A (**2**) and B (**3**), and six known compounds, 4′-hydroxyflavanone (**4**), apigenine (**5**), kampferol (**6**), luteolin (**7**), quercetin (**8**), and (-)-catechin (**9**). Their structures were determined by detailed analysis of spectroscopic data and comparison with the data of known analogues. Compounds **1** and **4**–**9** were evaluated for the inhibition of nitric oxide production in lipopolysaccharide (LPS)-stimulated product of nitrite in RAW 264.7 cells with IC_50_ values of 5.63, 15.72, 8.67, 6.67, 6.17, 7.61, and 14.52 μg/mL, respectively.

## 1. Introduction

**Figure 1:**
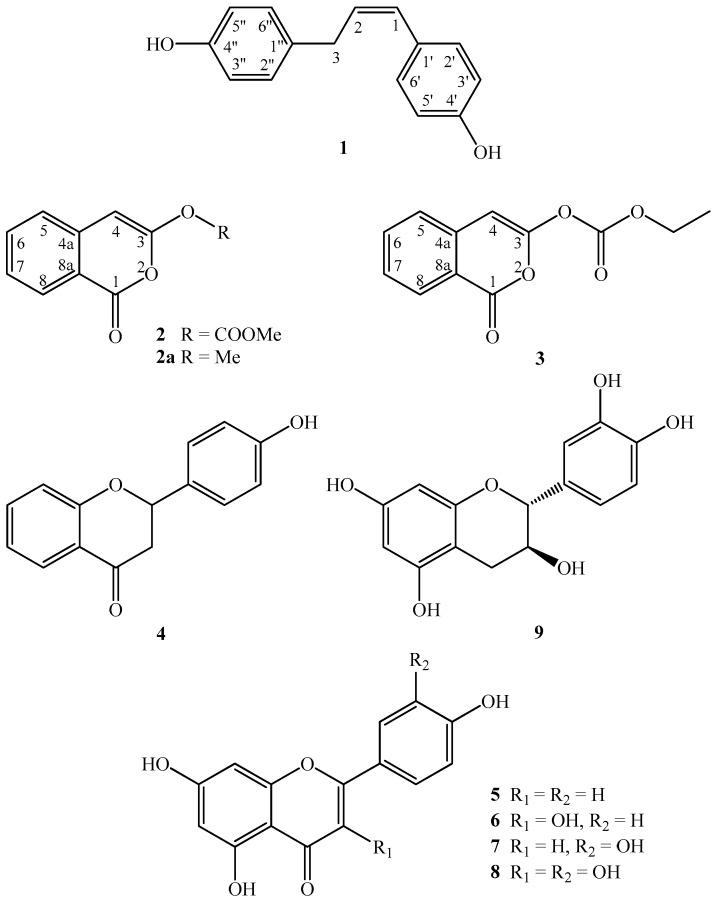
The chemical structures of compounds **1**–**9**.

*Lawsonia inermis* Linn (Lythraceae) is a tall shrub or small tree native to northern Africa, western and southern Asia, and northern Australasia [1]. *Lawsonia inermis* is a folk herbal medicine used in Taiwan for skin diseases and wounds [2]. Flavonoids, quinoids, naphthalene derivatives, triterpenoids, coumarins [3], and their derivatives are widely distributed in plants of the genus *Lythraceae*. Many of these compound derivatives exhibit anti-inflammatory [4], antibacterial, antifungal, antimycotic, and antiparasitic activities [5]. A continuing chemical investigation of the secondary metabolites of this plant resulted in the isolation and identification of a new diphenol, (*Z*)-4,4′-(prop-1-ene-1,3-diyl)diphenol (**1**), two new isocoumarin carbonates, inermiscarbonates A (**2**) and B (**3**) (Figure 1), as well as six flavonoids (**4**–**9**) from the *Lawsonia inermis*, and their structures are depicted in Figure 1. The isolation and detailed structural elucidation of the (*Z*)-4,4′-(prop-1-ene-1,3-diyl)diphenol (**1**), inermiscarbonates A (**2**), B (**3**), and the anti-inflammatory activities of all isolates are described herein.

## 2. Results and Discussion

### 2.1. Isolation and Structural Elucidation

The MeOH extract of the aerial part of *Lawsonia inermis* was concentrated to give a brown-green residue, which was suspended in water and partitioned with EtOAc and H_2_O, successively. The combined ethyl acetate (EtOAc)-soluble fraction was purified by repeated silica gel column chromatography and normal phase semipreparative high-performance liquid chromatography (HPLC) to obtain a new diphenol, (*Z*)-4,4′-(prop-1-ene-1,3-diyl)diphenol (**1**), two new isocoumarin carbonates, inermiscarbonates A (**2**) and B (**3**), and six known flavonoids, **4**–**9**. The identification of the known compounds was established through direct comparison with the published physical and spectral data.

**Figure 2:**
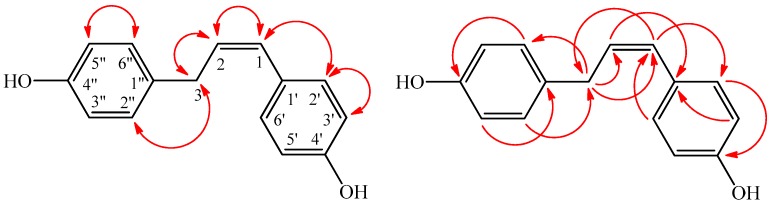
Significant NOESY (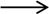) and HMBC (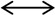) correlations of **1**.

Compound **1** was obtained as a pale yellow powder. Its molecular formula was calculated as C_15_H_14_O_2_ from the analysis of its high resolution electron spray mass spectrometry (HR-ESI-MS) data, corresponding to nine degrees of unsaturation. The UV spectrum exhibited conjugated absorption at λ (log ε) 285 (4.32) and 296 (4.24) nm. Its IR spectrum showed absorption bands for hydroxyl (3554 cm^−1^), olefinic (1652 cm^−1^), and aromatic (1602 and 1500 cm^−1^) functionalities. Two 1,4-disubstituted benzene rings were suggested from the ^1^H-NMR signals at δ_H_ 7.25 (2H, d, *J* = 8.5 Hz, H-2′, H-6′) and δ_H_ 6.73 (2H, d, *J* = 8.5 Hz, H-3′, H-5′), along with δ_H_ 7.04 (2H, d, *J* = 8.3 Hz, H-2″, H-6″) and δ_H_ 6.68 (2H, d, *J* = 8.3 Hz, H-3″, H-5″). The proton resonances of two olefinic methine (δ_H_ 6.00 (1H, dt, *J* = 10.5, 7.5 Hz, H-2: δ_C_ 142.6) and δ_H_ 5.70 (1H, d, *J* = 10.5 Hz, H-1: δ_C_ 110.5)) and one methylene (δ_H_ 3.57 (2H, d, *J* = 7.5 Hz, H-3)) indicated a partial structure of *cis*-double bond with an adjacent methylene group. The UV absorption at λ_max_ 285 and 296 nm with larger log ε value suggested the conjugated olefinic and phenyl groups. The presence of a 1-propenyl moiety linked between two phenyl moieties in **1** was predicted from the COSY correlations of H-2 (δ_H_ 6.00)/H-1 (δ_H_ 5.70) and H_2_-3 (δ_H_ 3.57)/H-2 (δ_H_ 6.00), as well as the HMBC correlations (Figure 2), as follows: H-1 (δ_H_ 5.70)/C-2′,6′ (δ_C_ 134.1), H-1 (δ_H_ 5.70)/C-3 (δ_C_ 36.8), H_2_-3 (δ_H_ 3.57)/C-1 (δ_C_ 110.5), and H_2_-3 (δ_H_ 3.57)/C-2 (δ_C_ 142.6) (Table 1). The connection of the two phenol groups (1,4-disubstituted benzene rings) was determined by the HMBC correlations of H-2″, H-6″ (δ_H_ 7.04)/C-3 (δ_C_ 36.8), H_2_-3 (δ_H_ 3.57)/C-2″, C-6″ (δ_C_ 130.6), and H-2′, H-6′ (δ_H_ 7.25)/C-1 (δ_C_ 110.5). Thus, compound **1** was assigned as (*Z*)-4,4′-(prop-1- ene-1,3-diyl)diphenol.

**Figure 3:**
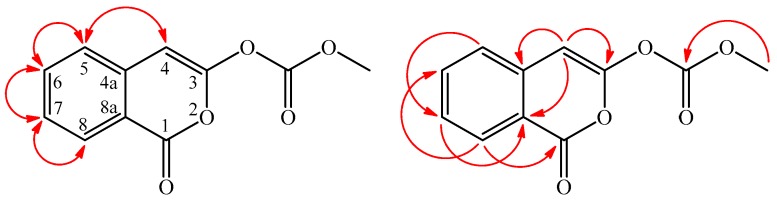
Significant NOESY (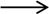) and HMBC (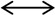) correlations of **2**.

Inermiscarbonate A (**2**) was isolated as an amorphous powder with molecular formula C_11_H_8_O_5_ as determined by positive-ion HR-ESI-MS, showing an [M + H]^+^ ion at *m*/*z* 221.1864 (calcd. for C_11_H_9_O_5_, 221.1861) and eight degrees of unsaturation. The IR spectrum demonstrated the presence of carbonate carbonyl (1793 cm^−1^) and isocoumarin carbonyl groups (1716 cm^−1^) [6]. The UV absorptions at *λ*_max_ (log ε) 244 (4.42), 255 (4.35), 272 (4.46), 281 (4.23), and 350 (3.11) nm indicated that compound **2** possessed isocoumarin with a 3-oxygenated skeleton as 3-methoxy-1*H*-isochromen-1-one (**2a**) [6]. The ^1^H- and ^13^C-NMR data of **2** were similar to those of 3-methoxy-1*H*-isochromen-1-one (**2a**) [7], except that the methyl carbonate group (δ_H_ 3.85 (3H, s, OCOCH_3_); δ_C_ 52.0 (OCOCH_3_), 164.1(OCOCH_3_)) at C-3 of **2** replaced the 3-methoxy group of **2a**. By the aid of HSQC, HMBC (Figure 3), ^1^H–^1^H COSY, and NOESY (Figure 3) techniques, and the full ^1^H- and ^13^C-NMR signals of **2** were unambiguously assigned (Table 2). Therefore, the structure of **2** was determined as methyl (4-oxo-1*H*-isochromen-3-yl)carbonate.

**Figure 4:**
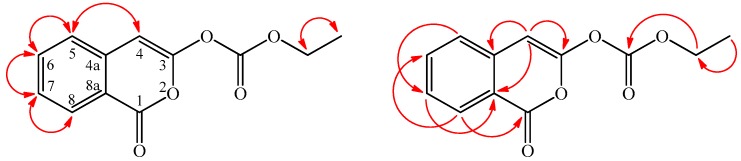
Significant NOESY (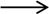) and HMBC (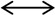) correlations of **3**.

Inermiscarbonate (**3**) was isolated as an amorphous powder. Compound **3** shows the molecular formula to be C_12_H_11_O_5_ due to the HR-ESI-MS molecular [M + H]^+^ ion at *m*/*z* 235.2129 (calcd. 235.2127), implying eight degrees of unsaturation. The IR spectrum demonstrated the presence of carbonate carbonyl (1772 cm^−1^) and isocoumarin carbonyl groups (1720 cm^−1^) [6]. The UV absorptions at λ_max_ (log ε) 244, 255, 272, 280, and 350 nm were similar to those of **2** and 3-methoxy-1*H*-isochromen-1-one (**2a**), and were characteristic of the 3-oxygenated isocoumarin skeleton [6]. The ^13^C-NMR spectrum of **3** showed signals for 12 carbons which were one more carbon signal than compound **2**. Comparison of the ^1^H-, ^13^C-NMR, and MS data of **3** with those of **2** suggested that their structures were closely related, except that the ethoxy group (δ_H_ 1.36 (t, *J* = 7.3 Hz, OCOCH_2_CH_3_), 4.30 (q, *J* = 7.3 Hz, OCOCH_2_CH_3_); δ_C_ 14.3 (OCOCH_2_CH_3_), 61.0 (OCOCH_2_CH_3_)) of **3** replaced the methoxy group of **2**. This was supported by HMBC correlation (Figure 4) between OCOCH_2_CH_3_ (δ_H_ 4.30) and OCOCH_2_CH_3_ (δ_C_ 163.6). The full assignment of ^1^H- and ^13^C-NMR resonances was supported by ^1^H–^1^H COSY, DEPT, HSQC, NOESY, and HMBC spectral analyses (Table 3). According to the above data, the structure of **3** was elucidated as ethyl (1-oxo-1*H*-isochromen-3-yl)carbonate. This is the first isolation of inermiscarbonate B from a natural source, although inermiscarbonate B was synthesized by Schnekenburger [8].

### 2.2. Structure Identification of the Known Isolates

The known isolates were readily identified by comparison of their physical and spectroscopic data (UV, IR, ^1^H-NMR, ${\left[\alpha \right]}_{D}^{25}$, and MS) with those of the corresponding authentic samples or literature values. They include six flavonoids: 4′-hydroxyflavanone (**4**) [9], apigenine (**5**) [10], kampferol (**6**) [11], luteolin (**7**) [10], quercetin (**8**) [12], and, (−)-catechin (**9**) [12]. 

### 2.3. Inhibitory Activity against Nitric Oxide Production

Nitric oxide (NO) is derived from the oxidation of l-arginine by NO synthase (NOS), and is a mediator in the inflammatory response involved in host defense [13]. In inflammation and carcinogenesis conditions, there is an increased production of NO by inducible NO synthase (iNOS) [14]. The anti-inflammatory effects of the compounds isolated from the *Lawsonia inermis* were also evaluated for the suppression of lipopolysaccharide (LPS)-induced NO generation in murine macrophage. In this study, the inhibitory activity of three new compounds (**1**–**3**) and six flavonoids (**4**–**9**) toward NO production was evaluated by the measurement of nitrite/nitrate in LPS-stimulated RAW 264.7 cells. To search for the appropriate concentrations for the above assay, these nine compounds were first tested for their cytotoxic activity against the RAW 264.7 cells, and no significant cytotoxic activities were observed under all tested concentrations. From the results of our anti-inflammatory tests, the following conclusions could be drawn: (a) The high cell viability (>92%) indicated that the inhibitory activities of compounds **1** and **4**–**9** on LPS-induced NO production did not result from their cytotoxicities; (b) Compounds **1**, **6**, and **7** exhibited inhibitory effects on lipopolysaccharide (LPS)-induced nitric oxide production in RAW 264.7 cells with IC_50_ values of 5.63, 6.67, and 6.17 μg/mL, respectively (Table 4). (c) (*Z*)-4,4′-(Prop-1-ene-1,3-diyl)diphenol (**1**) is the most effective among the isolated compounds against LPS-induced NO generation, with IC_50_ = 5.63 ± 3.64 μg/mL.

## 3. Experimental Section

### 3.1. General

UV spectra were obtained with a Shimadzu Pharmaspec-1700 UV-Visible spectrophotometer (Shimadzu, Kyoto, Japan). Infrared spectra were obtained with a Shimadzu IR prestige-21 Fourier transform (FT) infrared spectrophotometer (Shimadzu). 1D- and 2D-NMR spectra were recorded with a Bruker DRX-500 FT-NMR spectrometer (Bruker, Bremen, Germany). Mass spectrometric (HR-EI-MS) data were generated at the Mass Spectrometry Laboratory of the Chung Hsing University (Taichung, Taiwan). Column chromatography was performed using LiChroCART Si gel (5 μM; Merck, Darmstadt, Germany), and TLC analysis was carried out using aluminum pre-coated Si plates (Merck & Co., Inc.), and the spots were visualized using a UV lamp at λ = 254 nm.

### 3.2. Chemicals

The solvents used to open column isolation (Sephadex LH 20 and silica gel column) in the study, such as *n*-hexane, chloroform, ethyl acetate, acetone, and methanol were ACS grade. The HPLC grade *n*-hexane, ethyl acetate, and acetone for HPLC isolation, and the deuterated solvent for NMR measurement (CDCl_3_, CD_3_OD) were purchased from the branch of Merck in Taipei, Taiwan. LPS (endotoxin from *Escherichia coli*, serotype 0127:B8), Carr (type IV), indomethacin, MTT (3-(4,5-dimethylthiazol-2-yl)-2,5-diphenyltetrazolium bromide), and other chemicals were purchased from Sigma Chemical Co. (St. Louis, MO, USA).

### 3.3. Plant Material

*Lawsonia inermis* was collected from Neipu Township, Pingtung, Taiwan, in February 2009 and identified by I.-S. Chen (Emeritus Professor, School of Pharmacy, College of Pharmacy, Kaohsiung Medical University, Kaohsiung, Taiwan). A voucher specimen (CMU-LIY-090711) was deposited at the School of Chinese Pharmaceutical Sciences and Chinese Medicine Resources.

### 3.4. Extraction and Isolation

The dried aerial part (5.0 kg) of *Lawsonia inermis* was extracted three times with MeOH (50 L each) for 7 days. The extract was concentrated under reduced pressure at 35 °C, and the residue (440 g) was partitioned between EtOAc and H_2_O (1:1) to provide the EtOAc-soluble fraction (fraction A; 132.5 g). Fraction A (132.5 g) was purified by column chromatography (CC) (6.0 kg of SiO_2_, 70–230 mesh; *n*-hexane/EtOAc/methanol gradient) to afford 14 fractions: A1–A14.

Fraction A2 (32.8 g) was re-separated by silica gel column chromatography (*n*-hexane:ethyl acetate = 10:1) and semi-preparative normal phase HPLC (*n*-hexane:acetone = 8:1 to afford pure compounds **1** (37.5 mg), **2** (16.8 mg), and **3** (24.1 mg). Fraction 9 (16.45 g) was re-separated by Sephadex LH 20 column chromatography (chloroform:methanol = 3:7), silica gel column chromatography (*n*-hexane:ethyl acetate = 5:2), and then semi-preparative HPLC (*n*-hexane:acetone = 3:1) to afford pure compounds **4** (12.1 mg), **5** (8.5 mg), **6** (16.0 mg), **7** (14.6 mg), **8** (22.6 mg), and **9** (16.3 mg).

*(Z)-4,4′-(Prop-1-ene-1,3-diyl)diphenol* (**1**). Pale yellow powder; HR-ESI-MS: C_15_H_14_O_2_, found: 227.2787 [M + H]^+^, calcd.: 227.2784; UV (MeOH) λ_max_ (log ε): 285 (4.32), 296 (4.24) nm. IR (KBr) ν_max_: 3554 (OH), 1652, 1602, 1500, 1439 cm^−1^. ^1^H-NMR and ^13^C-NMR (500/125 MH_Z_, in CDOD) were shown on Table 1. 

*Methyl (1-oxo-1H-isochromen-3-yl)carbonate (Inermiscarbonate A)* (**2**). Amorphous powder; HR-ESI-MS: C_11_H_9_O_5_, found: 221.1864 [M + H]^+^, calcd.: 221.1861; UV (Dioxane) λ_max_ (log ε): 244 (4.42), 255 (4.35), 272 (4.46), 281 (4.23), 350 (3.11) nm. IR (KBr) ν_max_: 1793 (C=O), 1716 (C=O), 1599 cm^−1^. ^1^H-NMR and ^13^C-NMR (500/125 MH_Z_, in CDCl_3_) were shown on Table 2. 

*Ethyl (1-oxo-1H-isochromen-3-yl)carbonate (Inermiscarbonate B)* (**3**). Amorphous powder; HR-ESI-MS: C_12_H_11_O_5_, found: 235.2129 [M + H]^+^, calcd.: 235.2127; UV (Dioxane) λ_max_ (log ε): 244 (4.48), 255 (4.43), 272 (4.55), 280 (4.49), 350 (3.42) nm. IR (KBr) ν_max_: 1772 (C=O), 1720 (C=O), 1598 cm^−1^. ^1^H-NMR and ^13^C-NMR (500/125 MH_Z_, in CDCl_3_) were shown on Table 3. 

### 3.5. Cell Culture

A murine macrophage cell line RAW 264.7 (BCRC No. 60001) was purchased from the Bioresources Collection and Research Center (BCRC, Hsinchu, Taiwan) of the Food Industry Research and Development Institute (Hsinchu, Taiwan). Cells were cultured in plastic dishes containing Dulbecco’s Modified Eagle Medium (DMEM, Sigma) supplemented with 10% fetal bovine serum (FBS, Sigma) in a CO_2_ incubator (5% CO_2_ in air) at 37 °C and subcultured every 3 days at a dilution of 1:5 using 0.05% trypsin-0.02% EDTA in Ca^2+^-, Mg^2+^-free phosphate-buffered saline (DPBS).

### 3.6. Cell Viability

Cells (2 × 10^5^) were cultured in 96-well plates containing DMEM supplemented with 10% FBS for 1 day to become nearly confluent. Then cells were cultured with compounds **1**–**9** in the presence of 100 ng/mL LPS (lipopolysaccharide) for 24 h. After that, the cells were washed twice with DPBS and incubated with 100 μL of 0.5 mg/mL MTT for 2 h at 37 °C, testing for cell viability. The medium was then discarded, and 100 μL dimethyl sulfoxide (DMSO) was added. After 30-min incubation, absorbance at 570 nm was read using a microplate reader (Molecular Devices, Sunnyvale, CA, USA).

### 3.7. Measurement of Nitric Oxide/Nitrite

NO production was indirectly assessed by measuring the nitrite levels in the cultured media and serum determined by a colorimetric method based on the Griess reaction. The cells were incubated with different concentrations of samples in the presence of LPS (100 ng/mL) at 37 °C for 24 h. Then, cells were dispensed into 96-well plates, and 100 μL of each supernatant was mixed with the same volume of Griess reagent (1% sulfanilamide, 0.1% naphthylethylenediamine dihydrochloride and 5% phosphoric acid) and incubated at room temperature for 10 min, and the absorbance was measured at 540 nm with a Micro-Reader (Molecular Devices, SpectraMax^®^ M2e, Sunnyvale, CA, USA). By using sodium nitrite to generate a standard curve, the concentration of nitrite was measured from absorbance at 540 nm.

### 3.8. Statistical Analysis

The data is expressed as means ± standard errors (SE). The IC_50_ values were calculated from the dose curves using a non-linear regression algorithm (SigmaPlot 8.0; SPSS Inc., Chicago, IL, USA, 2002). Statistical evaluation was carried out by one-way analysis of variance (ANOVA followed by Scheffe’s multiple range tests).

## 4. Conclusions

Nine compounds, including three new compounds (**1**–**3**), were isolated from the aerial part of *Lawsonia inermis*. The structures of these compounds were established on the basis of spectroscopic data. New compounds **2** and **3** are carbonate derivatives, with very unique structures from natural sources. Compounds **1** and **4**–**9** showed effective anti-inflammatory activities by decreasing nitrate of LPS-stimulated production in RAW 264.7 cell with IC_50_ values of 5.63 ± 3.64 μg/mL 15.72 ± 2.52 μg/mL, 8.67 ± 3.84 μg/mL, 6.67 ± 3.48 μg/mL, 6.17 ± 2.86 μg/mL, 7.61 ± 3.34 μg/mL, and 14.52 ± 3.31 μg/mL, respectively, wherein compounds **2** and **3** exhibited no inhibition. (*Z*)-4,4′-(Prop-1-ene-1,3-diyl)diphenol (**1**) is a new compound and the most effective among the isolated compounds, with IC_50_ values of 5.63 ± 3.64 μg/mL against LPS-stimulated nitrite generation. Our study suggests *Lawsonia inermis* and its isolates (especially **1**, **6**, and **7**) could be further developed as potential candidates for the treatment or prevention of various inflammatory diseases.

## Figures and Tables

**Table 1:** NMR data (CD_3_OD) of **1**. δ in ppm, *J* in Hz.

Position	δ_C_	δ_H_	NOESY	HMBC ^a^
1	110.5	5.70 (d, *J* = 10.5 )	2, 2′, 6′	2, 3, 2′, 6′
2	142.6	6.00 (d, *J* = 10.5, 7.5)	1, 3	1′, 1″
3	36.8	3.57 (d, *J* = 7.5)	2, 2″, 6″	1, 2, 2″, 6″
1′	129.0			
2′, 6′	134.1	7.25 (d, *J* = 8.5)	1, 3′, 5′	1, 4′
3′, 5′	116.7	6.73 (d, *J* = 8.5)	2′, 6′	1′
4′	157.7			
1″	132.2			
2″, 6″	130.6	7.04 (d, *J* = 8.3)	3, 3″, 5″	3, 4″
3″, 5″	116.4	6.68 (d, *J* = 8.3)	2″, 6″	1″
4″	156.8			

^a^ From the H- to the C-atom.

**Table 2:** NMR data (CDCl_3_) of **2**. δ in ppm, *J* in Hz.

Position	δ_C_	δ_H_	NOESY	HMBC ^a^
1	165.5			
2				
3	154.1			
4	95.6	5.88 (s)	5	3, 4a, 8a
4a	138.9			
5	126.0	7.69 (dd, *J* = 7.1, 1.5)	4, 6	4, 7, 8a
6	135.0	7.74 (td, *J* = 7.1, 1.7)	5, 7	8, 4a
7	121.2	7.78 (td, *J* = 7.1, 1.5)	6, 8	5, 8a
8	132.3	7.99 (dd, *J* = 7.1, 1.7)		1, 6, 4a
8a	124.7			
OCOR	164.1			
OCOCH_3_	52.0	3.85 (s)		OCOCH_3_

^a^ From the H- to the C-atom.

**Table 3:** NMR data (CDCl_3_) of **3**. δ in ppm, *J* in Hz.

Position	δ_C_	δ_H_	NOESY	HMBC ^a^
1	165.5			
2				
3	154.0			
4	96.0	5.88 (s)	5	3, 4a, 8a
4a	139.0			
5	126.0	7.69 (dd, *J* = 7.1, 1.5)	4, 6	4, 7, 8a
6	135.0	7.77 (td, *J* = 7.1, 1.7)	5, 7	8, 4a
7	121.2	7.74 (td, *J* = 7.1, 1.5)	6, 8	5, 8a
8	132.3	7.98 (dd, *J* = 7.1, 1.7)		1, 6, 4a
8a	124.8			
OCOR	163.6			
OCOCH_2_CH_3_	61.0	4.30 (q, *J* = 7.3)	OCOCH_2_CH_3_	OCOCH_2_CH_3_
OCOCH_2_CH_3_	14.3	1.36 (t, *J* = 7.3)	OCOCH_2_CH_3_	OCOCH_2_CH_3_

^a^ From the H- to the C-atom.

**Table 4:** Inhibitory effect of compounds **1**–**9** on overproduction of nitric oxide in lipopolysaccharide (LPS)-stimulated RAW 264.7 cells.

Compounds	IC_50_ (μg/mL) ^a^
**1**	5.63 ± 3.64
**2**	>20
**3**	>20
**4**	15.72 ± 2.52
**5**	8.67 ± 3.84
**6**	6.67 ± 3.48
**7**	6.17 ± 2.86
**8**	7.61 ± 3.34
**9**	14.52 ± 3.31
Indomethacin ^b^	78.56 ± 1.38

^a^ The IC_50_ values were calculated from the slope equation of the dose–response curves. Values are expressed as mean S.E.M. of three independent experiments. ^b^ Indomethacin was used as a positive control.

## Supplementary Materials

Supplementary materials can be accessed at: http://www.mdpi.com/1420-3049/21/10/1299/s1.

## References

[1] Chen H.Y., Qian C. (2007). Flora of China.

[2] Lin Y.X., Chang Y.S., Chen I.S., Ou J.C. (2003). The Catalogue of Medicinal Plant Resources in Taiwan.

[3] Ahmed S., Rahman A., Alam A., Saleem M., Athar M., Sultana S. (2000). Evaluation of the efficacy of *Lawsonia alba* in the alleviation of carbon tetrachloride-induced oxidative stress. J. Ethnopharmacol..

[4] Liou J.R., Mohamed E.S., Du Y.C., Tseng C.N., Hwang T.L., Chuang Y.L., Hsu Y.M., Hsieh P.W., Wu C.C., Chen S.L. (2013). 1,5-Diphenylpent-3-en-1-ynes and methyl naphthalene carboxylates from *Lawsonia inermis* and their anti-inflammatory activity. Phytochemistry.

[5] Babu P.D., Subhasree R.S. (2009). Antimicrobial activities of *Lawsonia inermis*—A review. Acad. J. Plant Sci..

[6] Neuman J.R.C., Behar J.V. (1967). 2-Carbomethoxybenzocyclobutenone. Synthesis of a photochemically sensitive small-ring system by a pyrolytic Wolff rearrangement. J. Am. Chem. Soc..

[7] Sevil Ö., Metin B. (2008). The chemistry of homophthalic acid: A new synthetic strategy for construction of substituted isocoumarin and indole skeletons. Tetrahedron.

[8] Schnekenburger J. (1965). Acylierung von Homophthalsäureanhydrid mit kohlensäureäthylesterchlorid. J. Arch. Pharm..

[9] Kagawa H., Shigematsu A., Ohta S., Harigaya Y. (2005). Preparative monohydroxyflavanone syntheses and a protocol for gas chromatography-mass spectrometry analysis of monohydroxyflavanones. Chem. Pharm. Bull..

[10] Parveen S., Riaz N., Saleem M., Khan J., Ahmad S., Ashraf M., Ejaz S.A., Tareen R.B., Jabbar A. (2012). Bioactive phenolics from *Launaea intybacea*. J. Chem. Soc. Pak..

[11] Liu M., Yang S., Jin L., Hu D., Wu Z., Yang S. (2012). Chemical constituents of the ethyl acetate extract of *Belamcanda chinensis* (L.) DC roots and their antitumor activities. Molecules.

[12] Qi S.H., Wu D.G., Ma Y.B., Luo X.D. (2003). A novel flavane form *Carapa guianensis*. Acta Bot. Sin..

[13] Geller D.A., Billiar T.R. (1998). Molecular biology of nitric oxide synthases. Cancer Metastasis Rev..

[14] Moncada S., Palmer R.M., Higgs E.A. (1991). Nitric oxide: Physiology, pathophysiology, and pharmacology. Pharmcol. Rev..

