# Supplementary material for: New Diphenol and Isocoumarins from the Aerial Part of Lawsonia inermis and Their Inhibitory Activities against NO Production
Source: Molecules. 2016 Sep 28;21(10):1299. doi: 10.3390/molecules21101299 (PMC6273290; doi:10.3390/molecules21101299)
Supplement: Supplementary file 1 [file molecules-21-01299-s001.pdf]

Supplementary Materials: New Diphenol and Isocoumarins from the Aerial Part of *Lawsonia inermis* and their Inhibitory Activities against NO Production

Chang-Syun Yang, Hui-Chi Huang, Sheng-Yang Wang, Ping-Jyun Sung, Guan-Jhong Huang, Jih-Jung Chen, and Yueh-Hsiung Kuo

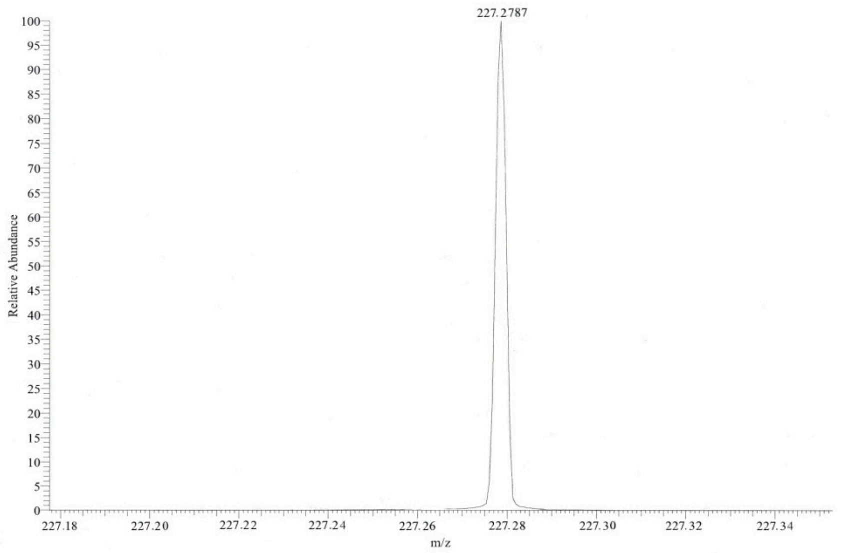

Figure S1. HR-ESI-MS spectrum of **1**.

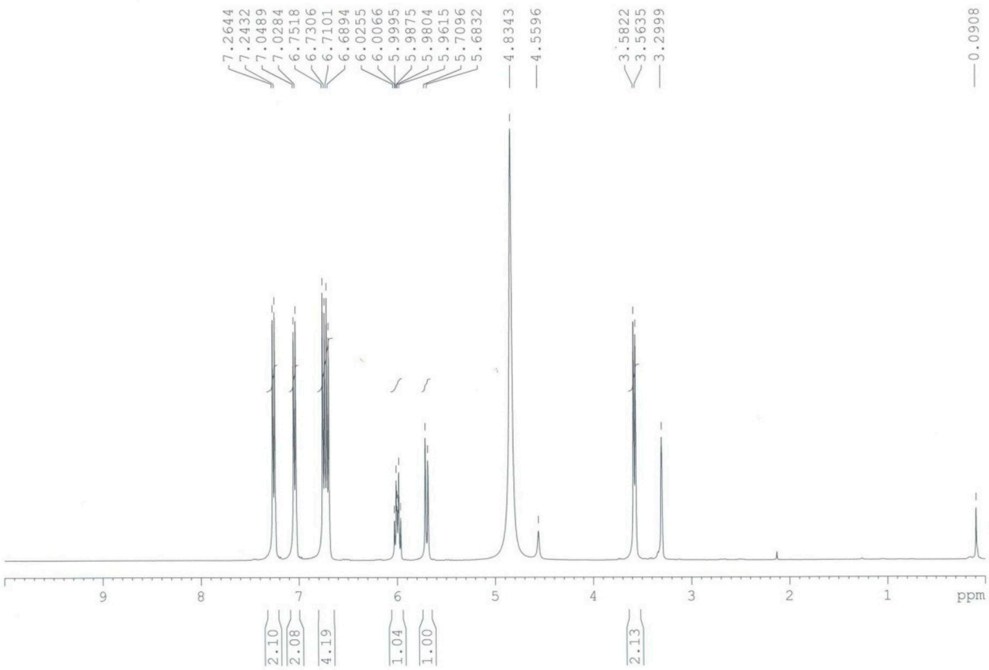

Figure S2. ^{1}H-NMR spectrum of **1** (CD_3OD, 500 MHz).

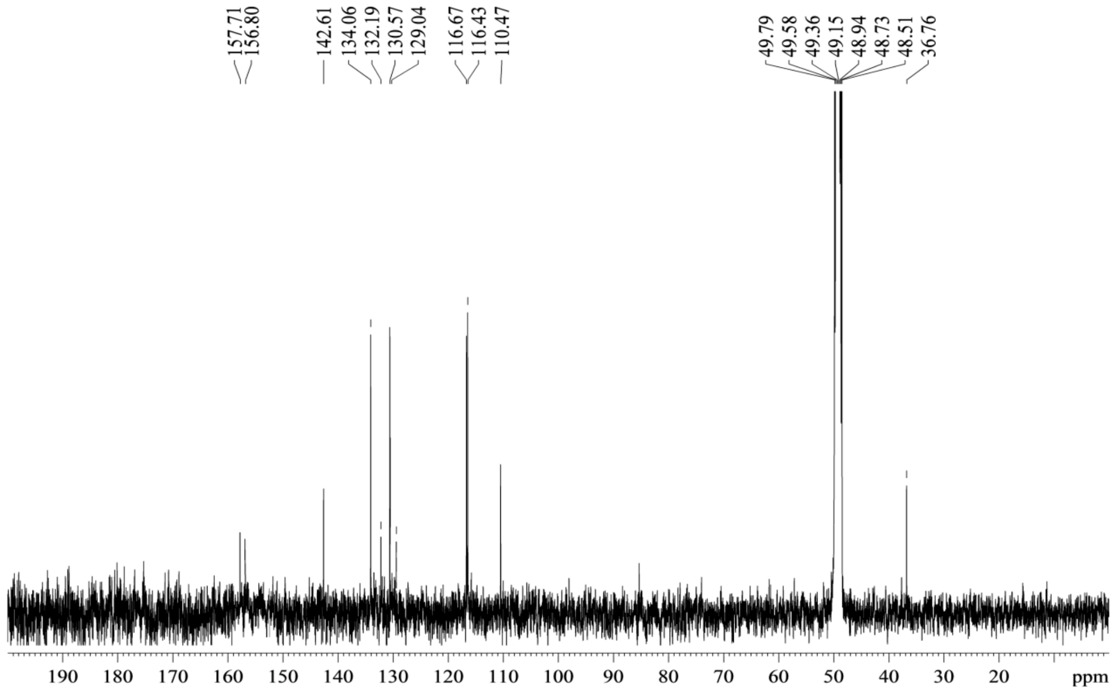

Figure S3. ^{13}C-NMR spectrum of **1** (CD_3OD, 125 MHz).

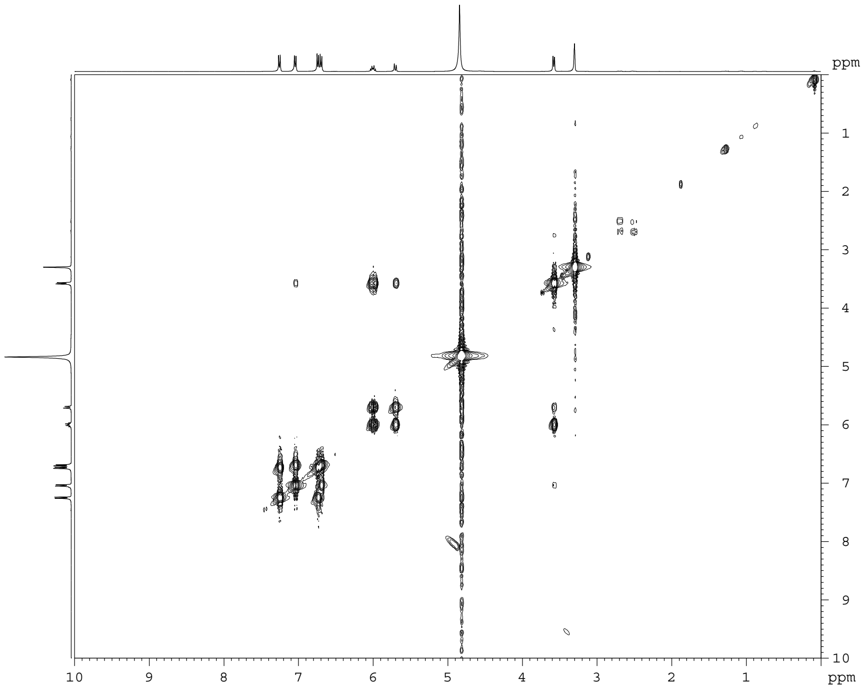

Figure S4. ^{1}H–^{1}H COSY spectrum of **1**.

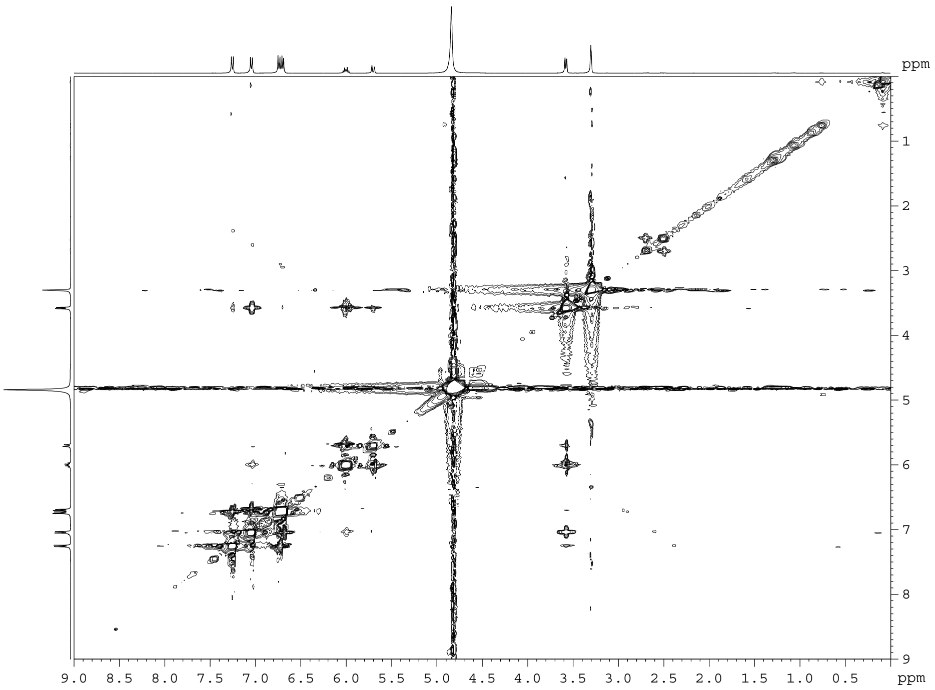

Figure S5. NOESY spectrum of **1**.

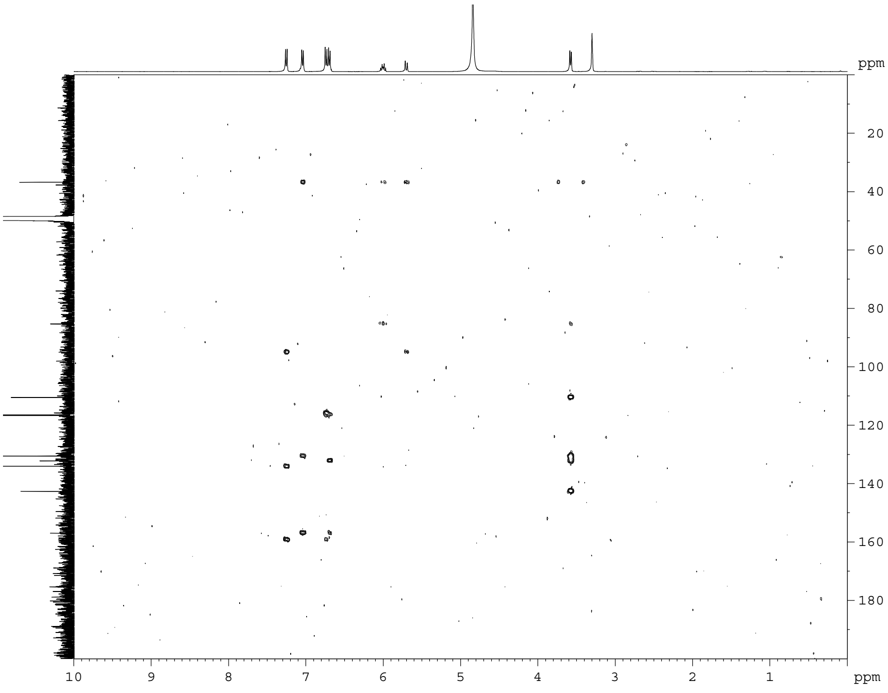

Figure S6. HMBC spectrum of **1**.

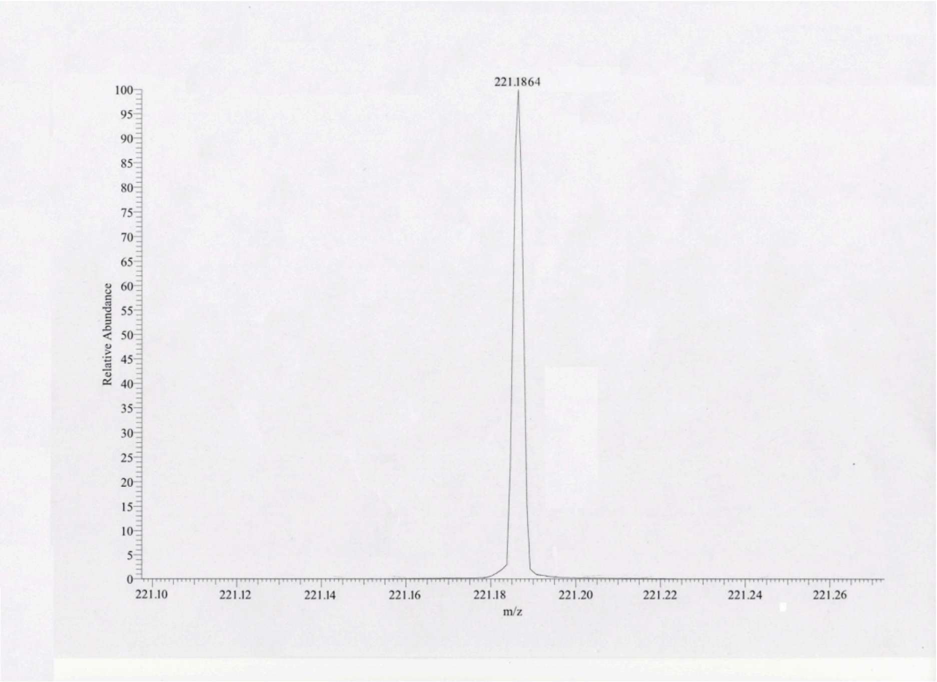

Figure S7. HR-ESI-MS spectrum of **2**.

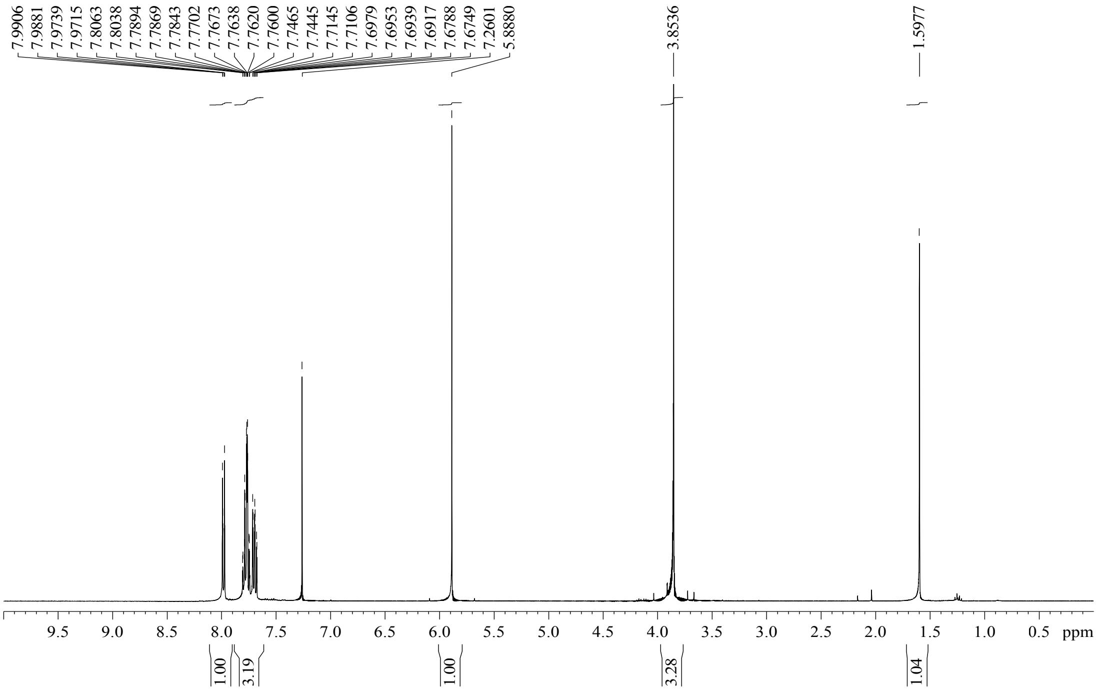

Figure S8. ¹H-NMR spectrum of **2** ($CDCl_3$, 500 MHz).

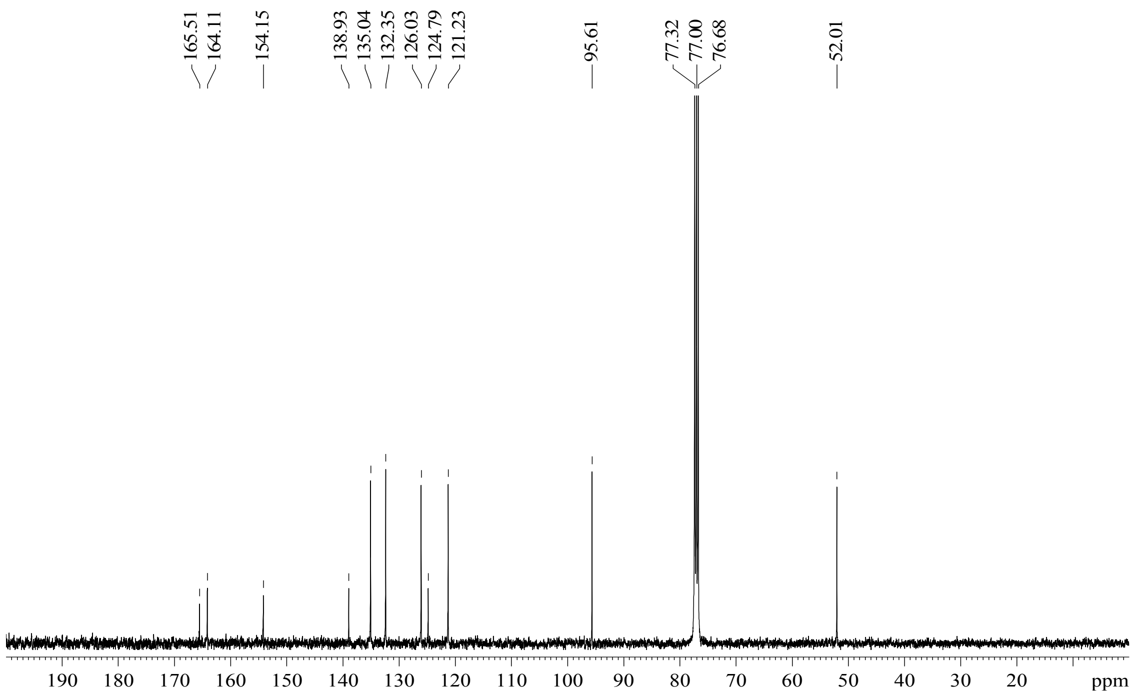

Figure S9. ^{13}C-NMR spectrum of **2** ($CDCl_3$, 125 MHz).

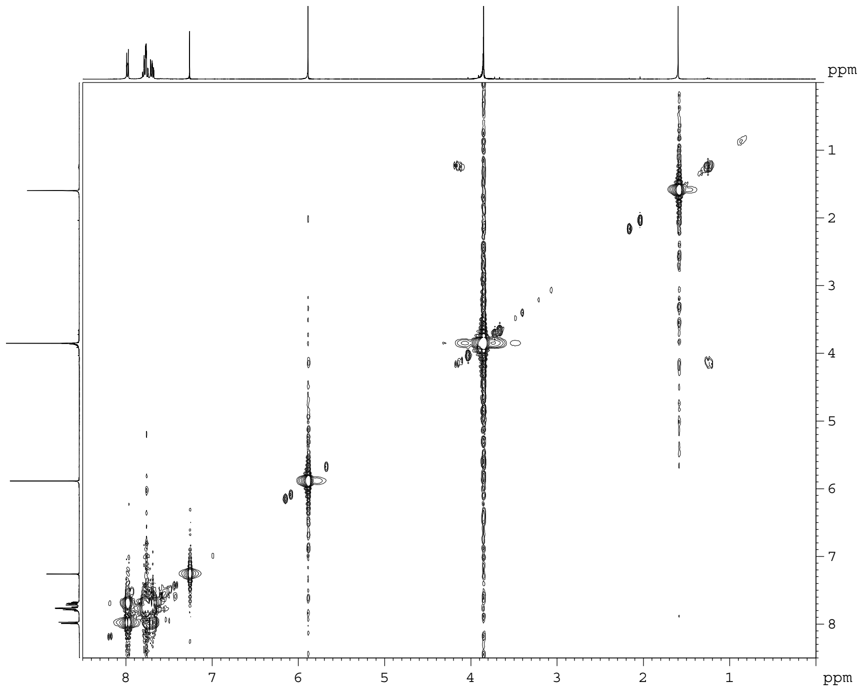

Figure S10. ^{1}H–^{1}H COSY spectrum of **2**.

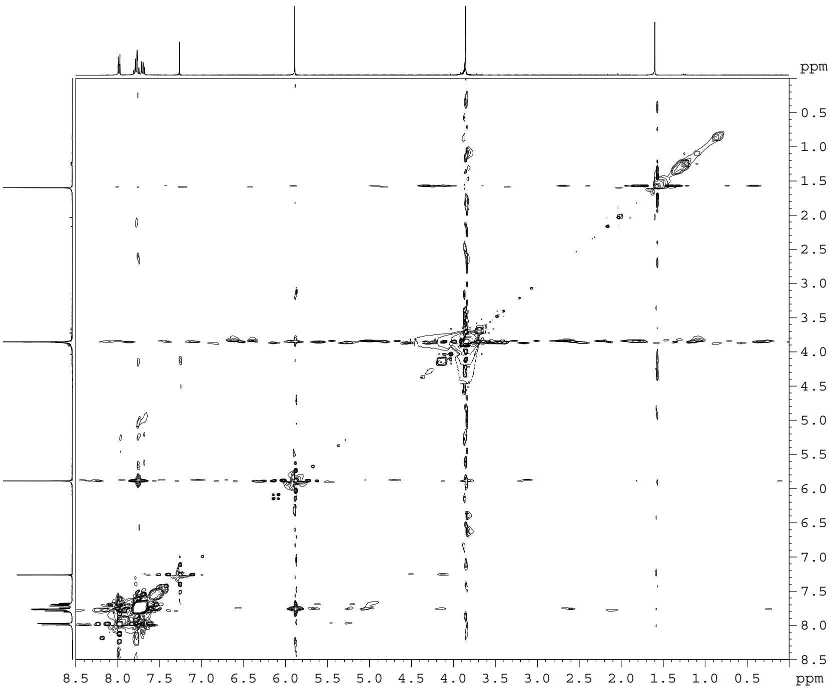

Figure S11. NOESY spectrum of **2**.

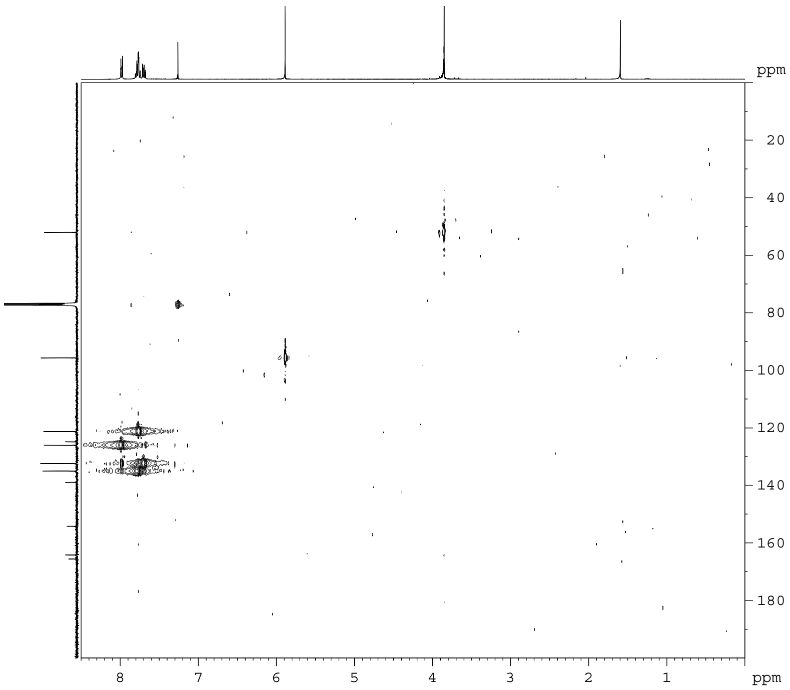

Figure S12. HMBC spectrum of **2**.

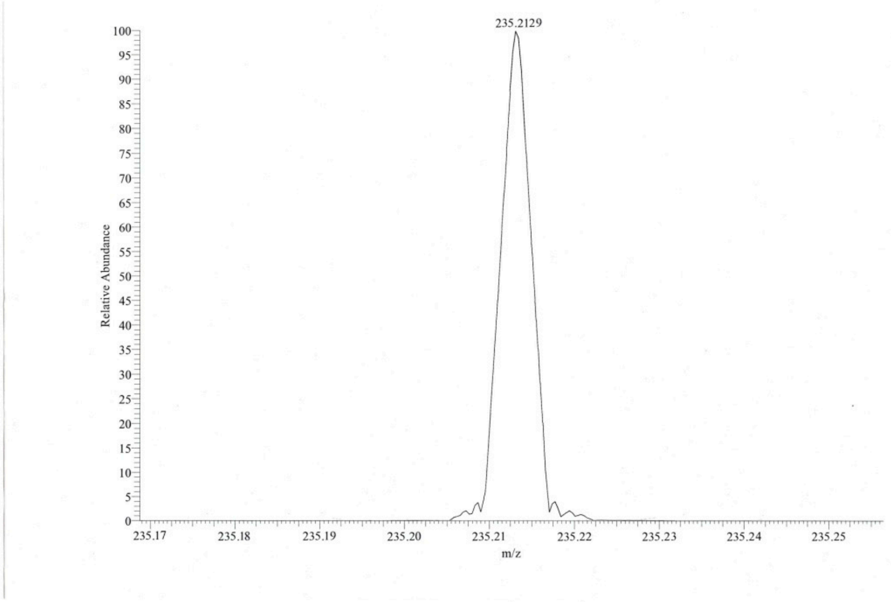

Figure S13. HR-ESI-MS spectrum of **3**.

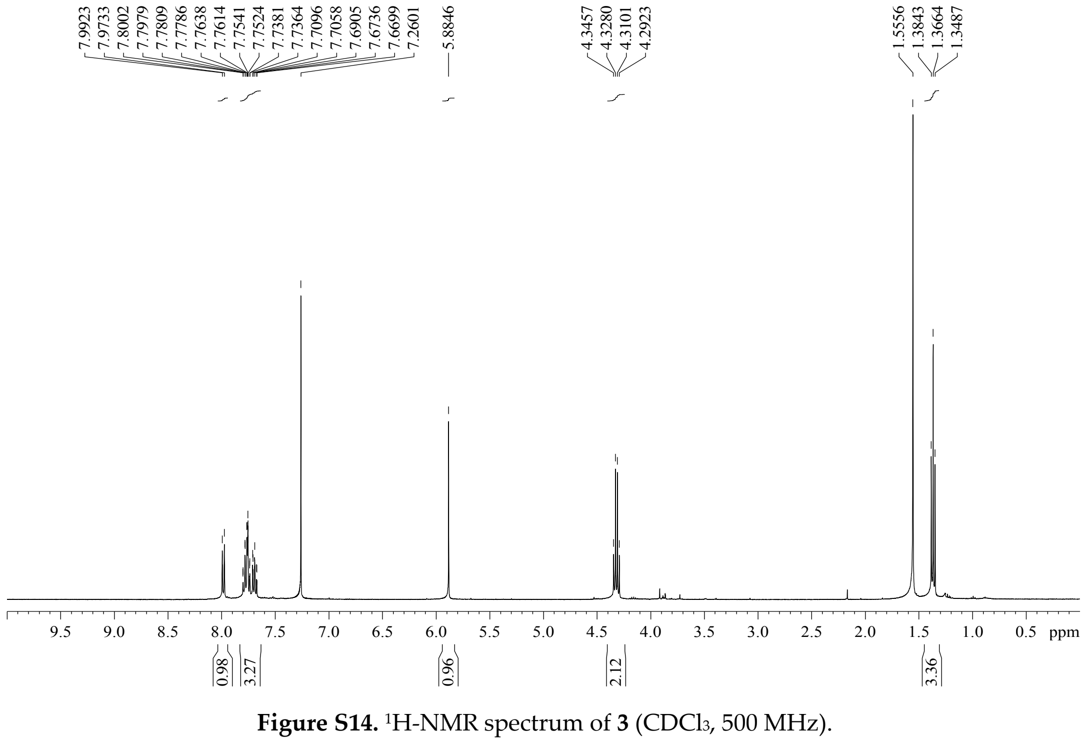

Figure S14. ^{1}H-NMR spectrum of **3** ($CDCl_3$, 500 MHz).

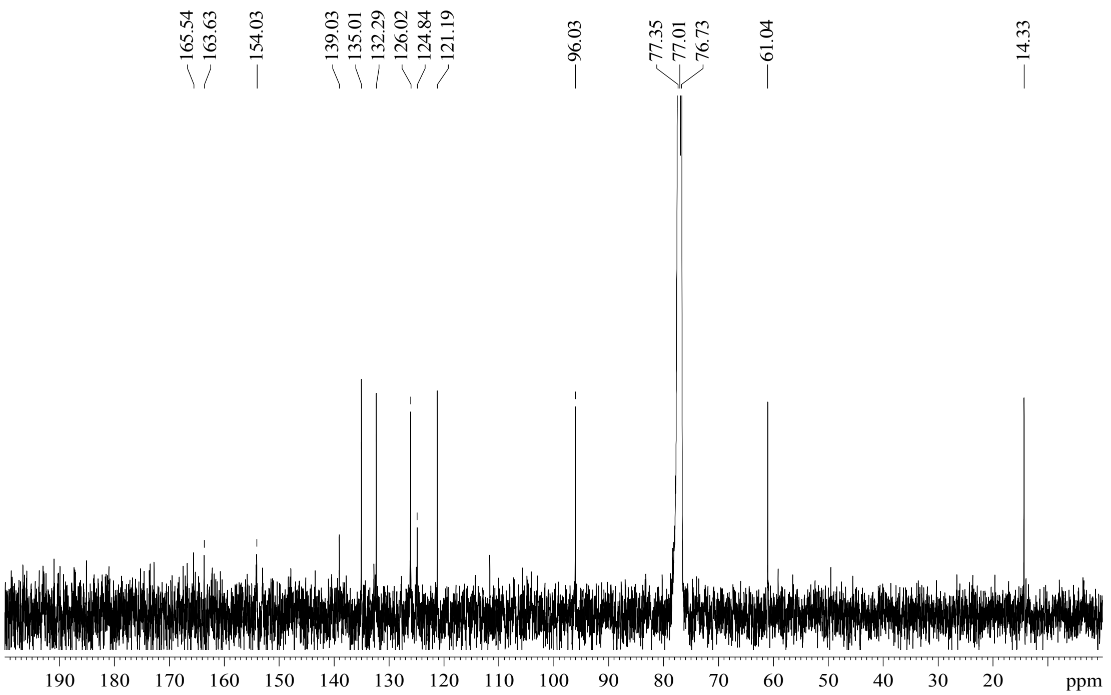

Figure S15. ^{13}C-NMR spectrum of **3** ($CDCl_3$, 125 MHz).

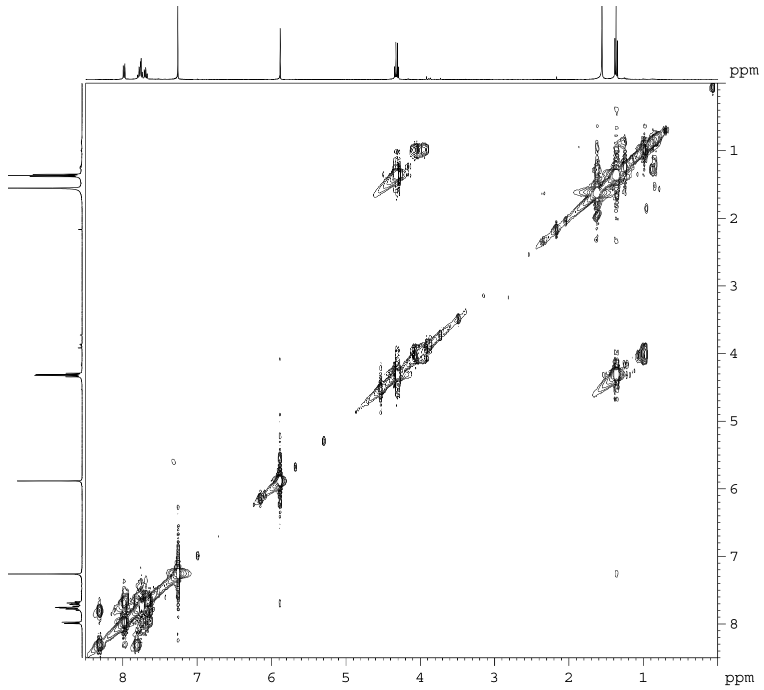

Figure S16. ^1H–^1H COSY spectrum of **3**.

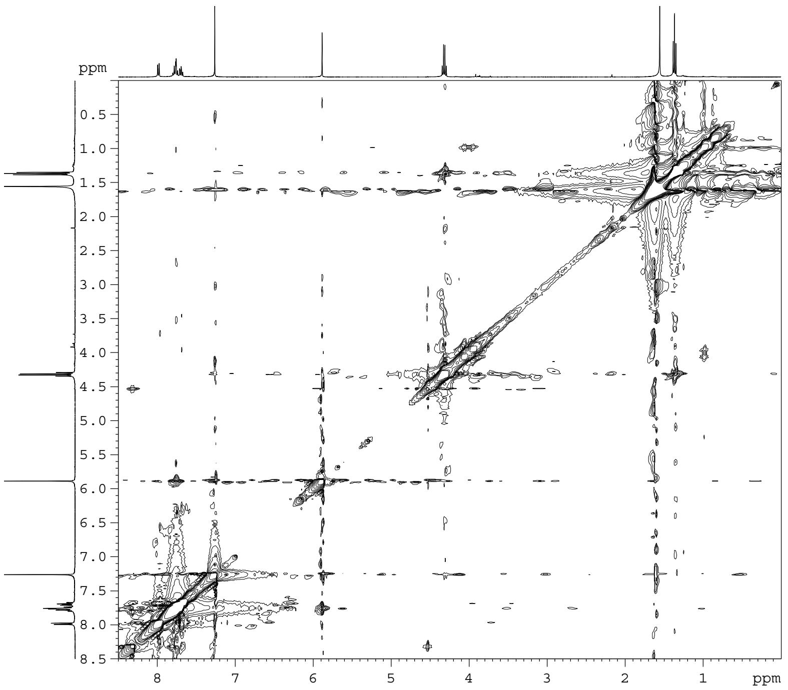

Figure S17. NOESY spectrum of **3**.

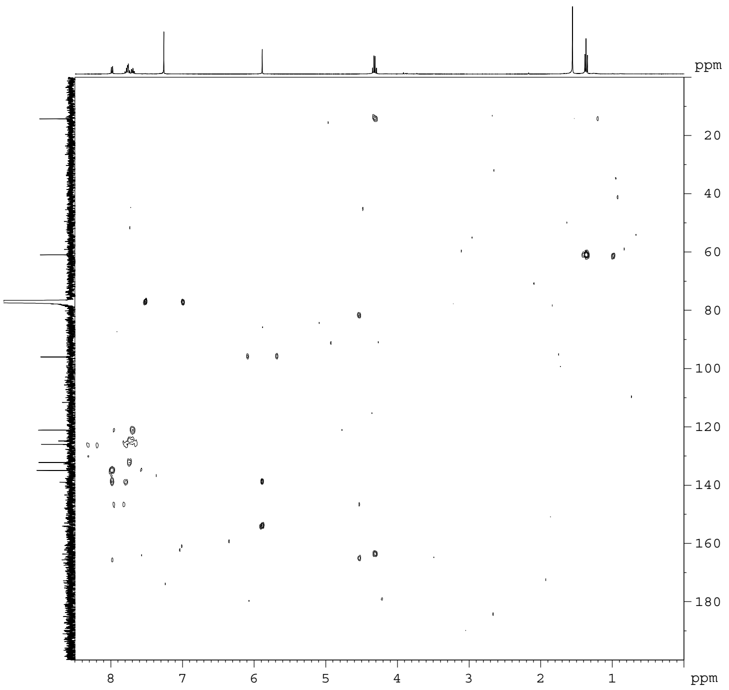

Figure S18. HMBC spectrum of **3**.

Table S1. Inhibitory effect of compounds **1–9** on overproduction of nitric oxide in LPS-stimulated RAW 264.7 cells.

	**Dose (μM/mL)**	**Cell Viability (% of Control)**	**NO Inhibition (% of Control)**	**IC_{50} (μg/mL)**
Control	(-)	92.8 ± 6.1	(-)	
LPS	(+)	99.5 ± 2.3	(-)	
(*Z*)-4,4′-(Prop-1-ene-1,3-diyl)diphenol (**1**)	2.5	95.0 ± 1.0	38.8 ± 4.0	5.63 ± 3.64
	5	87.6 ± 1.5	47.8 ± 4.7	
	10	78.5 ± 1.5	60.2 ± 1.8	
	20	58.4 ± 2.2	81.6 ± 2.9	
Inermiscarbonates A (**2**)	2.5	98.8 ± 1.4	5.7 ± 0.7	>20
	5	87.0 ± 1.9	6.4 ± 0.7	
	10	83.1 ± 1.7	2.3 ± 0.5	
	20	79.2 ± 2.4	1.6 ± 0.5	
Inermiscarbonates B (**3**)	2.5	99.7 ± 1.6	-4.8 ± 0.7	>20
	5	100.4 ± 1.9	0.4 ± 0.5	
	10	95.0 ± 1.4	9.7 ± 0.6	
	20	83.0 ± 1.1	28.0 ± 0.5	
4′-Hydroxyflavanone (**4**)	2.5	99.7 ± 2.3	8.2 ± 0.3	15.72 ± 2.52
	5	95.8 ± 2.8	14.0 ± 0.7	
	10	93.4 ± 2.6	32.0 ± 2.9	
	20	67.1 ± 2.1	59.6 ± 1.4	
Apigenine (**5**)	2.5	93.4 ± 1.3	-11.1 ± 0.5	8.67 ± 3.84
	5	93.7 ± 1.1	32.4 ± 2.7	
	10	81.4 ± 1.5	54.6 ± 3.4	
	20	62.6 ± 1.5	78.2 ± 2.4	
Kampferol (**6**)	2.5	97.9 ± 1.2	30.7 ± 1.5	6.67 ± 3.48
	5	92.0 ± 0.7	41.1 ± 3.8	
	10	77.0 ± 1.6	62.5 ± 2.6	
	20	59.3 ± 1.2	75.9 ± 0.7	
Luteolin (**7**)	2.5	99.3 ± 2.3	30.9 ± 2.4	6.17 ± 2.86
	5	100.7 ± 1.7	46.0 ± 1.8	
	10	96.1 ± 1.2	59.1 ± 3.2	
	20	80.1 ± 1.8	81.6 ± 1.8	
Quercetin (**8**)	2.5	100.8 ± 1.6	23.6 ± 1.6	7.61 ± 3.34
	5	100.2 ± 1.8	40.8 ± 3.5	
	10	98.6 ± 1.4	56.0 ± 3.6	
	20	92.2 ± 0.9	66.7 ± 3.5	
(−)-Catechin (**9**)	2.5	89.4 ± 2.5	20.7 ± 2.9	14.52 ± 3.31
	5	83.4 ± 1.4	28.8 ± 1.1	
	10	80.2 ± 2.3	41.2 ± 1.2	
	20	78.6 ± 2.9	57.5 ± 0.8	
